# Influence of composition of cysteine-containing peptide-based chelators on biodistribution of ^99m^Tc-labeled anti-EGFR affibody molecules

**DOI:** 10.1007/s00726-018-2571-1

**Published:** 2018-05-04

**Authors:** Maryam Oroujeni, Ken G. Andersson, Xenia Steinhardt, Mohamed Altai, Anna Orlova, Bogdan Mitran, Anzhelika Vorobyeva, Javad Garousi, Vladimir Tolmachev, John Löfblom

**Affiliations:** 1 Department of Immunology, Genetics and Pathology, Uppsala University, 75181 Uppsala, Sweden; 2 Department of Protein Science, KTH-Royal Institute of Technology, Stockholm, Sweden; 3 Department of Medicinal Chemistry, Uppsala University, Uppsala, Sweden

**Keywords:** Affibody molecules, EGFR, ^99m^Tc, Peptide-based chelators, Glutamate

## Abstract

Epidermal growth factor receptor (EGFR) is overexpressed in a number of cancers and is the molecular target for several anti-cancer therapeutics. Radionuclide molecular imaging of EGFR expression should enable personalization of anti-cancer treatment. Affibody molecule is a promising type of high-affinity imaging probes based on a non-immunoglobulin scaffold. A series of derivatives of the anti-EGFR affibody molecule ZEGFR:2377, having peptide-based cysteine-containing chelators for conjugation of ^99m^Tc, was designed and evaluated. It was found that glutamate-containing chelators Gly-Gly-Glu-Cys (GGEC), Gly-Glu-Glu-Cys (GEEC) and Glu-Glu-Glu-Cys (EEEC) provide the best labeling stability. The glutamate containing conjugates bound to EGFR-expressing cells specifically and with high affinity. Specific targeting of EGFR-expressing xenografts in mice was demonstrated. The number of glutamate residues in the chelator had strong influence on biodistribution of radiolabeled affibody molecules. Increase of glutamate content was associated with lower uptake in normal tissues. The ^99m^Tc-labeled variant containing the EEEC chelator provided the highest tumor-to-organ ratios. In conclusion, optimizing the composition of peptide-based chelators enhances contrast of imaging of EGFR-expression using affibody molecules.

**Electronic supplementary material:**

The online version of this article (10.1007/s00726-018-2571-1) contains supplementary material, which is available to authorized users.

## Introduction

Aberrant expression of growth-controlling receptors is a part of cellular phenotypes for several kinds of cancer. Specific targeting of these cancer-associated abnormalities is a promising strategy in treatment of disseminated malignancies. The epidermal growth factor receptor (EGFR or HER1) is a transmembrane tyrosine kinase receptor that normally regulates cell proliferation, suppression of apoptosis, and motility. EGFR is overexpressed in a number of malignant tumors, like carcinomas of head and neck, breast, urinary bladder and lung. Interruption of EGFR signaling by blocking ligand binding sites on the extracellular domain or by inhibiting intracellular tyrosine kinase activity can hinder the growth of EGFR-expressing tumors (Mendelsohn and Baselga 2006; Scaltriti and Baselga 2006). Overexpression of EGFR predicts responses of head and neck and lung cancers to several treatments (Bentzen et al. 2005; Hirsch et al. 2006; Pirker et al. 2012). Analysis of biopsy samples is currently the common method to determine the level of EGFR expression in tumors. However, the use of biopsies is associated with several problems, such as high level of heterogeneity, difference of EGFR expression in primary tumors and metastases (Scartozzi et al. 2004), sensitivity and specificity of antibodies (Kersting et al. 2006) and changes of EGFR expression during therapy (Choong et al. 2007). The use of radionuclide molecular imaging of EGFR expression can overcome many of these problems (Pantaleo et al. 2009). The unmet clinical need for sensitive detection of EGFR expression in disseminated malignancies has encouraged the development of radionuclide imaging probes based on monoclonal antibodies (Divgi et al. 1991; Menke-van der Houven et al. 2015; Makris et al. 2015), antibody fragments (van Dijk et al. 2016) and scaffold proteins (Kruziki et al. 2016; Garousi et al. 2017). One of the issues when imaging this particular target is the noticeable expression of EGFR in hepatocytes. This may cause sequestering of the imaging probe in liver and prevent or decrease its accumulation in tumors (Divgi et al. 1991). Nevertheless, adjustment of amount of injected targeting protein has enabled partial saturation of the receptors on hepatocytes and thereby allows radiolabeled probes to pass the “liver barrier” (Divgi et al. 1991; Tolmachev et al. 2010; Miao et al. 2012).

Affibody molecules are currently evaluated as targeting moieties for radionuclide molecular imaging (Ståhl et al. 2017). Affibody molecules (6–7 kDa) are robust three-helical proteins developed using the scaffold of the 58-amino acid-long Z domain of staphylococcal protein A. Affibody molecules are highly soluble and can refold with high fidelity after thermal or chemical denaturation (Arora et al. 2004), which permits the use of harsh labeling condition and expands the number of applicable labeling methods. Moreover, these molecules show binding to tumor-associated target proteins with high affinities in the picomolar to low nanomolar range, which is crucial for high retention in the tumors. Their small size makes it possible to obtain higher-contrast images in comparison to bulky monoclonal antibodies (150-kDa). Combination of small size, high affinity and specificity makes affibody molecules suitable ligands for both molecular imaging and therapeutic applications (Lofblom et al. 2010; Tolmachev et al. 2007; Ståhl et al. 2017). Clinical and preclinical studies have indicated that affibody molecules can provide high sensitivity and specificity for visualizing several targets in cancer xenografts, e.g. human epidermal growth factor 2 (HER2), human epidermal growth factor receptor type 3 (HER3), insulin-like growth factor 1 receptor (IGF-1R), platelet-derived growth factor receptor β (PDGFRβ) and carbonic anhydrase IX (CAIX) using single photon emission computed tomography (SPECT) and positron emission tomography (PET) techniques (Ståhl et al. 2017).

**Fig. 1:**
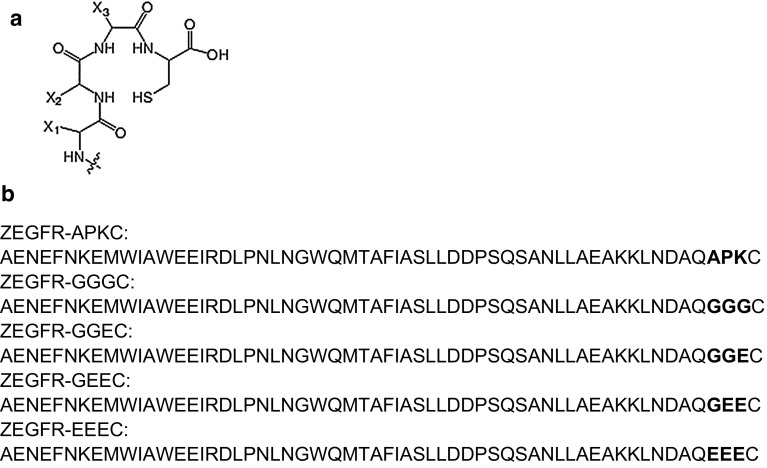
Peptide-based chelators used for labeling with ^99m^Tc. **a** General structure of the N_3_S chelators formed by a C-terminal cysteine and three adjacent amino acids. X_1_, X_2_ and X_3_ denote the side chains of the amino acids. **b** Sequences of EGFR-binding affibody molecules evaluated in this study. The variable amino acids are marked in bold

^99m^Tc (*T*_1/2_ = 6 h) is the most commonly used radionuclide in nuclear medicine. Excellent availability due to generator production, favorable emitted photon energy (Eγ = 140.5 keV), and low absorbed-dose burden to the patient are the advantages of this radionuclide (Banerjee et al. 2005). However, development of appropriate labeling chemistry is an important precondition for the use of a radionuclide as a label for any application. The radionuclides must remain stably attached to the targeting probe in vivo to prevent the dissociation of free radionuclides in blood and healthy tissues, leading to increased background signal. Since ^99m^Tc shows a high thiol-affinity, it may be chelated by short peptide sequences incorporating a thiol-containing moiety. In such structure, amide nitrogens of adjacent amino acids form together with the thiol group of the cysteine a N_3_S chelator, providing a stable complex with ^99m^Tc (Fig. 1a). Earlier, several derivatives of the anti-HER2 ZHER2:342 and ZHER2:2395 affibody molecules have been stably labeled using cysteine-containing peptide-based chelators (Ahlgren et al. 2009; Altai et al. 2012). The use of a peptide-based cysteine-containing chelator for coupling of ^99m^Tc to an affibody molecule would offer two advantages. First, the chelating moiety would be incorporated during recombinant production, and no conjugation of a chelator is required. Second, modification of amino acid composition of such chelator might enable modulation of the biodistribution properties of the tracer.

Recently, we have demonstrated that addition of a cysteine to the C-terminus of the anti-EGFR affibody molecule ZEGFR:2377 forms a peptide-based cysteine-containing chelator suitable for labeling with ^99m^Tc (Andersson et al. 2016). That study demonstrated feasibility of imaging of EGFR expression in tumors using affibody molecules labeled using aforementioned methodology. However, further improvement of imaging contrast is desirable in order to enhance sensitivity of imaging. Studies using HER2-targeting affibody molecules demonstrated that it is possible to modulate the residualizing properties of the ^99m^Tc label, its retention in excretory organs and the predominant excretion pathway by varying the composition of the peptide-based chelator (Wållberg et al. 2011; Altai et al. 2012). This permitted to obtain excellent contrast of imaging. It would be attractive to apply the same methodology to increase the imaging contrast of EGFR-targeted affibody molecules as well. The current study was dedicated to testing the hypothesis that modification of cysteine-containing peptide-based chelators improves the biodistribution and imaging properties of EGFR-targeting affibody molecules.

When designing a panel of affibody molecules for this study, we took into account the results from the study reported by Andersson and co-workers (2016) that indicated that some amino acids in ZEGFR:2377 form a chelating site, which competes with the cysteine-containing chelator, but has appreciably lower stability of a complex with the nuclide. The presence of such chelating site would be associated with the loss of the site-specificity of the labeling. However, using a more stable chelator for labeling would potentially result in preferential complexing of the radiometal at the intended site. Earlier studies suggested that peptide-based chelators containing glutamic acid were that most stable in the case of anti-HER2 affibody molecules (Tran et al. 2007). Therefore, this study was focused on glutamate-based chelators.

A panel of engineered anti-EGFR affibody molecules was created, containing the same amino acid sequence at the N-terminus (AEN–), as well as APKC, GGGC, GGEC, GEEC and EEEC peptide-based chelators (Fig. 1b) at the C-terminus. Labeling of these affibody molecules with ^99m^Tc was optimized, and their stability, in vitro and in vivo targeting properties were investigated.

## Materials and methods

^99m^Tc was obtained as pertechnetate by elution of Ultra-TechneKow generator (Mallinckrodt) with sterile 0.9% sodium chloride (Mallinckrodt, Petten, The Netherlands). The radioactivity was measured using a gamma-spectrometer with a NaI(Tl) detector (1480 WIZARD, Wallac Oy, Turku, Finland).

The EGFR-specific affibody molecule, ZEGFR:2377, was produced and purified using ion-exchange chromatography and RP-HPLC as previously described (Andersson et al. 2016). For protein characterization, purity was measured using RP-HPLC (Zorbax 300SB-C18; Agilent Technologies, Inc., Palo Alto, CA, USA) using a gradient from 30–50% or 40–50% of 0.1% trifluoroacetic acid in acetonitrile for 35 min at a flow rate of 1 mL/min. Mass was determined using MALDI-TOF (SCIEX, Stockholm, Sweden), and the melting temperature was measured using circular dichroism spectroscopy (Jasco Inc., Easton, MD, USA). Affinity against recombinant human EGFR was analyzed in duplicates using single cycle kinetics in a Biacore T200 (GE Healthcare, Uppsala, Sweden).

### Labeling and in vitro stability test

Labeling and stability tests were performed according to previously described methods (Andersson et al. 2016). The C-terminally engineered affibody molecules were site-specifically labeled with ^99m^Tc using a lyophilized kit. Lyophilized labeling kits containing 75 µg of tin (II) chloride dihydrate (Fluka Chemika, Buchs, Switzerland), 5 mg of gluconic acid sodium salt (Celsus Laboratories, Geel, Belgium) and 100 µg of EDTA (Sigma-Aldrich, Munich, Germany) were prepared for labeling of affibody molecules with ^99m^Tc as described earlier (Ahlgren et al. 2010).

Two different protocols (A and B) were used for labeling. In Protocol A, 50 µg of affibody molecule in PBS (50 µL) was mixed with one lyophilized labeling kit and 100 μL of ^99m^Tc-generator eluate (typically, 500–800 MBq) was added. The mixture was thoroughly vortexed and incubated at 90 °C for 1 h. The ^99m^Tc-labeled conjugate was isolated using size-exclusion chromatography on a disposable NAP-5 column, pre-equilibrated and eluted with PBS.

In Protocol B, cysteine treatment of ^99m^Tc-labeled affibody molecule before purification was used to remove loosely bound ^99m^Tc. The labeling with ^99m^Tc, i.e. reduction of pertechnetate and transchelation of technetium to affibody molecules, was performed as described in Protocol A. After the labeling, a freshly prepared solution of cysteine (1 mg/mL in PBS, 300-fold molar excess to affibody molecules) was added to the reaction mixture and incubated at 90 °C for 15 min. After the cysteine challenge ^99m^Tc-labeled protein was isolated using a NAP-5 column.

The in vitro stability of ^99m^Tc-labeled affibody molecules was evaluated under four conditions: PBS, in the presence of 300-fold molar excess of cysteine (cysteine challenge) (Hnatowich et al. 1994), and in the presence of ascorbic acid tin (II) chloride to prevent oxidation by air oxygen (Andersson et al. 2016). For this purpose, samples of labeled and purified affibody molecules (2.2 µg, 50 µL) were mixed with either cysteine (12 µg, 1 mg/mL in PBS), sodium ascorbate (13 µg, 1 mg/mL in PBS) or SnCl_2_ × 2H_2_O (10 µg, 1 mg/mL in 0.01 M HCl). Control samples were mixed with 12 µL PBS. The samples were incubated at 37 °C and the radiochemical purity was measured by radio-ITLC at 1, 2 and 4 h. The experiment was performed in triplicate.

Radiolabeling yield and radiochemical purity of ^99m^Tc-labeled proteins were measured using instant thin-layer chromatography (ITLC) (ITLC-SG, Agilent Technologies, US) in PBS (affibody molecules: *R*_f_ = 0.0, other forms of ^99m^Tc *R*_f_ = 1.0). The reduced hydrolyzed technetium colloid (RHT) level was measured using pyridine: acetic acid: water (10:6:3) as a mobile phase (^99m^Tc colloid *R*_f_ = 0.0, other forms of ^99m^Tc and radiolabeled affibody molecule *R*_f_ = 1.0). The results of ITLC analysis were confirmed by a sodium dodecyl sulfate polyacrylamide gel electrophoresis (SDS-PAGE) using NuPAGE 4–12% Bis–Tris gel in MES buffer. A sample of ^99m^Tc-pertechnetate was used as a reference standard for low molecular weight compounds. Both reference and ^99m^Tc-labeled affibody molecule samples were run in parallel on the same gel at 200 V during 30 min. The distribution of radioactivity on ITLC strips and SDS-PAGE gels was measured by Cyclone Storage Phosphor System (Perkin-Elmer, Wellesley, MA, USA).

### In vitro studies

For cell studies, three EGFR-expressing cell lines with different level of EGFR expression, A431 (epidermoid carcinoma), MDA468 (breast carcinoma) and PC3 (prostate carcinoma) (ATCC) were used. The EGFR expression level is 1.2 × 10^6^, 1.6 × 10^6^ and 3 × 10^4^ receptors per cell for A431, MDA486 and PC3, respectively (Yang et al. 2001). The cell lines were cultured in McCoy’s medium, supplemented with 10% fetal bovine serum (Sigma-Aldrich), 1% l-glutamine, and PEST (penicillin 100 µ/mL and 100 µg/mL streptomycin), all from Bookroom AG (Berlin, Germany). The cells were cultured at 37 °C in a humidified incubator with 5% CO_2_.

For a binding specificity test, cells were seeded to culture dishes to obtain approximately 10^6^ cells/dish at the time of the experiments. To a set of control dishes, a 50-fold molar excess of anti-EGFR antibody, cetuximab, was added before addition of the labeled affibody molecules to saturate EGFR. Cells were incubated for 1 h at 37 °C with 10 nM ^99m^Tc-labeled affibody conjugate. After incubation, the medium was aspirated. Thereafter, the cells were washed with cold serum free-medium, treated with 0.5 mL trypsin–EDTA solution per dish at 37 °C and collected. Radioactivity of cells was measured and percentage of cell-bound radioactivity was calculated. The experiments were performed in triplicate.

To investigate the affinity of the labeled conjugates to EGF receptors, kinetics of binding of ^99m^Tc-conjugates to and their dissociation from A431 cells were measured using a LigandTracer yellow instrument (Ridgeview Instruments AB, Vänge, Sweden). The measurements were performed at room temperature to prevent internalization. Uptake curves were recorded at 0.33, 1 and 3 nM of the labeled conjugate, thereafter the radioactive medium was withdrawn, fresh medium was added and the dissociation curve was recorded. The data was analyzed using the Interaction Map software (Ridgeview Diagnostics AB, Uppsala, Sweden) to calculate association rate, dissociation rate and dissociation constant at equilibrium (K_D_). Analysis was performed in duplicates.

Cellular processing of bound ^99m^Tc-ZEGFR conjugates was evaluated using A431 cell. The cells were incubated with 10 nM the labeled conjugate at 37 °C. At 1, 2, 4, 6 and 24 h after incubation start, the internalized fraction was determined by acid washings. A group of three dishes was removed from the incubator, the media was collected and cells were washed two times with ice-cold serum-free medium. Thereafter, cells were treated with 1 mL 0.2 M glycine buffer, pH 2, containing 4 M urea, for 5 min at room temperature. The acidic solution was collected and cells were additionally washed with 1 mL glycine buffer. Thereafter cells were treated with 1.5 mL of PBS. This fraction was collected and cells were additionally washed with 1.5 mL PBS. Finally, cells were treated with 1.5 mL of phosphate buffer pH 8 for 15 min at room temperature. This fraction was collected and cells were washed additionally with 1.5 mL phosphate buffer. To measure the internalized conjugate, the cells were incubated with 1 mL 1 M NaOH at 37 °C for 10 min. The cell debris was collected, and the dishes were additionally washed with 1 mL of NaOH solution. The alkaline solutions were pooled. The radioactivity in the acidic solution, PBS and phosphate buffer solutions were considered as total membrane radioactivity bound, and in the alkaline fractions as internalized, respectively.

### In vivo studies

Animal experiments were approved by the Local Ethics Committee for Animal Research in Uppsala. Biodistribution studies were performed in female BALB/C nu/nu mice purchased from Taconic M&B a/S (Ry, Denmark). For each conjugate, 12 mice, randomized into groups of four, were used. EGFR-expressing xenografts were established by subcutaneous injection of 10^7^ A431 cells in the right hind legs of mice. The experiments were performed 12–14 days after cell implantation. The in vivo specificity of conjugates was investigated using saturation of EGF receptors in a control group of mice by subcutaneous injection of 10 mg of cetuximab 48 h before the injection of radiolabeled affibody molecules. Then, two groups of mice (including control group) were intravenously injected with each conjugate (38 µg of affibody molecule, 140 kBq in 100 µL PBS per mouse) for biodistribution measurement at 6 h after injection and one group of mice was injected with 1 MBq (38 µg of affibody molecule) for measurement of biodistribution at 24 h after injection. The injected protein dose was determined in earlier studies as an optimal for a partial saturation of EGFR in liver without saturation of receptors in tumors (Tolmachev et al. 2010). The mice were euthanized by an intraperitoneal injection of anesthesia (Ketalar 10 mg/mL, Rompun 1 mg/mL; 20 mL/g body weight) overdose at 6 and 24 h after injection. Blood and salivary glands, lung, liver, spleen, stomach, colon, kidneys, tumor, muscle and bone were collected and weighed. Then, the organ radioactivity was measured and uptake values of organs were calculated as percent injected activity per gram tissue (% ID/g).

### SPECT/CT imaging of EGFR-expressing xenografts using ^99m^Tc affibody molecules

Mice bearing A431 tumors were injected via the tail vein with 40 µg (30 MBq) of ^99m^Tc-ZEGFR-GGEC, ^99m^Tc-ZEGFR-GEEC or ^99m^Tc-ZEGFR-EEEC. Whole body SPECT/CT scans were performed at 6 h and 24 h p.i. using nanoScan SC (Mediso Medical Imaging Systems, Hungary) under sevoflurane anesthesia. CT acquisitions were carried out using CT-energy peak of 50 keV, 670 μA, 480 projections, and 2.29 min acquisition time. SPECT helical scans were acquired at the following parameters: ^99m^Tc energy window (126.45–154.56 keV), 110 projections, 256 × 256 matrix. The scan time was adjusted to 1 h for the 6 h pi time point. At the later time point of 24 h, the mice were scanned for 2 h for better counting statistics. CT raw files were reconstructed using Nucline 2.03 Software (Mediso Medical Imaging Systems, Hungary). SPECT raw data were reconstructed using Tera-Tomo™ 3D SPECT.

### Statistics and data treatment

Statistical treatment and linear regression analysis were performed using GraphPad Prism software version 5.00 for Windows, GraphPad Software, San Diego California. The difference was considered as significant when *P* value was less than 0.05.

## Results

The affibody molecules were successfully produced and purified using ion-exchange chromatography and a RP-HPLC polishing step as previously reported by Andersson and co-workers (2016). Measured protein size was in agreement with the theoretical size (Table 1; Supplemental Fig. 1) and the melting temperature of each construct was estimated to 50–37 °C (Table 1). A reduction in thermostability was observed for each consecutive decrease in isoelectric point of the constructs (Table 1). Protein purity was determined to ≥ 97% for all of the produced proteins (Supplemental Fig. 2) and the affinity to recombinant human EGFR was measured to low nanomolar affinity (Table 1).

**Table 1:** Protein characterization

Construct	Estimated size (da)	Measured size (da)	Isoelectric point (pI)	Tm (°C)	Measured purity (%)	Association rate constant (10^4^ 1/Ms)	Dissociation rate constant (10^−4^ 1/s)	Affinity K_D_ (nM)
ZEGFR-APKC	6692	6686	4.07	50	>99	2.4 ± 0.05	1.8 ± 0.1	7.3 ± 0.6
ZEGFR-GGGC	6566	6580	3.90	47	97	1.50 ± 0.01	1.8 ± 0.4	11 ± 0.4
ZEGFR-GGEC	6638	6639	3.84	44	>99	2.10 ± 0.01	4.3 ± 0.9	21 ± 0.6
ZEGFR-GEEC	6710	6705	3.79	42	>99	2.80 ± 0.01	7.6 ± 1.9	26 ± 0.5
ZEGFR-EEEC	6783	6783	3.75	37	97	2.1 ± 0.0	1.0 ± 0.0	21 ± 0

### Labeling and in vitro stability test

**Fig. 2:**
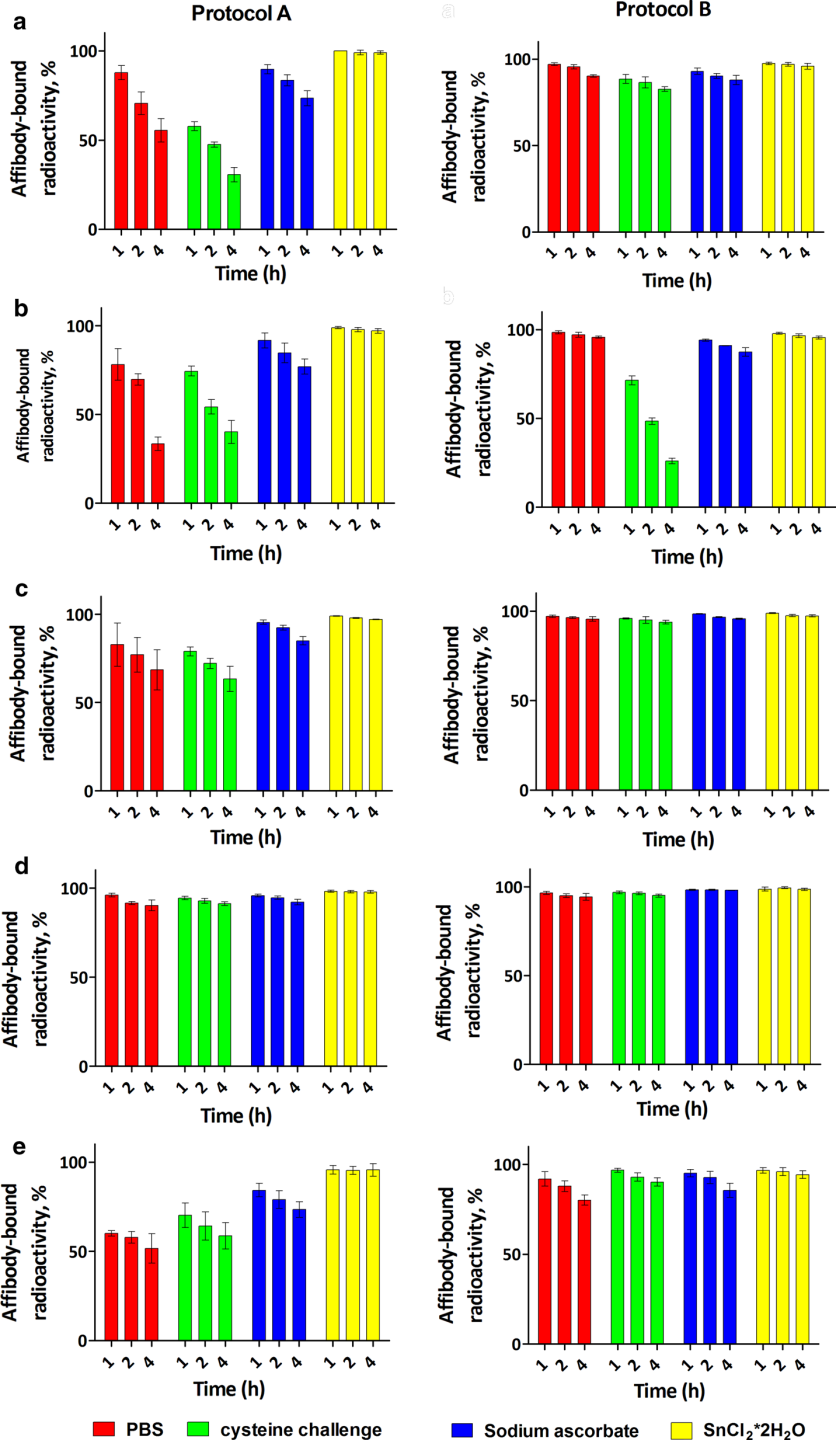
In vitro stability of ^99m^Tc-ZEGFR conjugates after labeling using different protocols: **a** ^99m^Tc-ZEGFR-APKC, **b** ^99m^Tc-ZEGFR-GGGC; **c** ^99m^Tc-ZEGFR-GGEC, **d** ^99m^Tc-ZEGFR-GEEC; **e** ^99m^Tc-ZEGFR-EEEC. Protocol B included pre-purification cysteine challenge. Data present the affibody-bound radioactivity after incubation in PBS (red), PBS containing 300-fold molar excess of cysteine (green), PBS containing sodium ascorbate (blue) and PBS containing tin (II) chloride (yellow). The data are presented as average (n ≥ 3) and SD

Initially, the labeling was performed according to Protocol A, i.e. without an intermediate cysteine challenge before purification. The radiochemical purity of the labeled affibody molecules after purification using NAP-5 columns was above 95% for all the five conjugates (Table 2). However, the results of the in vitro stability test (Fig. 2) demonstrated rapid release of ^99m^Tc not only under cysteine challenge, but also during storage in PBS for all variants except ZEGFR-GEEC. Interestingly, when we added sodium ascorbate to prevent re-oxidation by air, the release of radioactivity decreased. A similar effect was observed when we added stannous chloride as an antioxidant.

**Table 2:** Labeling of affibody molecules according to Protocol A (no cysteine challenge before purification)

	Radiochemical yield^a^ (%)	Isolated yield^b^ (%)	Radiochemical purity (%)	Maximum apparent specific activity^c^ (MBq/µg)
ZEGFR-APKC	98 ± 1	52 ± 2	100 ± 0	5.6
ZEGFR-GGGC	96 ± 0	83 ± 1	100 ± 0	6.2
ZEGFR-GGEC	99 ± 0.0	77 ± 1	99 ± 0.0	6.4
ZEGFR-GEEC	93 ± 2	79 ± 1	98 ± 1	11.7
ZEGFR-EEEC	73 ± 6	64 ± 1	95 ± 1	3.5

^a^Radiochemical yield is determined as percentage of affibody-bound activity before purification as measured by ITLC

^b^Isolated yield is determined as percentage of activity in the high molecular weight fraction after NAP-5 purification

^c^Maximum apparent specific activity obtained at the end of purification

We, therefore, introduced an intermediate cysteine challenge before purification (Protocol B) to remove loosely bound ^99m^Tc. The radiochemical yield of all variants after cysteine challenge was reduced (Table 3). ^99m^Tc-ZEGFR-APKC showed the lowest radiochemical yield (16 ± 1%) and isolated yield values (15 ± 2%), while ^99m^Tc-ZEGFR-GGEC and ^99m^Tc- ZEGFR-GEEC showed the highest radiochemical yield and isolated yield values after purification. The in vitro stability results showed that the release of ^99m^Tc was appreciably reduced after cysteine pre-challenge. Still, the stability of APKC and GGGC chelators was unsatisfactory but glutamate-containing variants demonstrated reasonable stability under dilution and under cysteine challenge. Based on labeling and stability results, we proceeded to continue in vitro and in vivo studies with ZEGFR-GGEC, ZEGFR-GEEC and ZEGFR-EEEC.

**Table 3:** Labeling of affibody molecules according to Protocol B (with pre-purification cysteine challenge)

	Radiochemical yield after cysteine challenge^a^	Isolated yield^b^ (%)	Radiochemical purity (%)	Maximum apparent specific activity^c^ (MBq/µg)
ZEGFR-APKC	16 ± 1	15 ± 2	98 ± 1	1.9
ZEGFR-GGGC	42 ± 3	34 ± 1	99 ± 0	2.6
ZEGFR-GGEC	84 ± 4	68 ± 3	100 ± 0	9.14
ZEGFR-GEEC	85 ± 5	69 ± 3	98 ± 1	9.1
ZEGFR-EEEC	55 ± 8	51 ± 6	96 ± 2	5.8

^a^Radiochemical yield is determined as percentage of affibody-bound activity after pre-purification cysteine challenge but before purification as measured by ITLC

^b^Isolated yield is determined as percentage of activity in the high molecular weight fraction after NAP-5 purification

^c^Maximum apparent specific activity obtained at the end of purification

The SDS-PAGE analysis showed no indication of aggregation or release of ^99m^Tc-pertechnetate, since no radioactivity bands of higher or lower molecular weight could be observed (Supplemental Fig. 3).

### In vitro studies

Binding specificity test (Supplemental Fig. 4) demonstrated that the binding of all ^99m^Tc-labeled conjugates to EGFR-expressing cell lines A431, MDA468, as well as PC3 cells, was receptor mediated, since saturation of the receptors with a large excess of anti-EGFR antibody cetuximab significantly (*P* < 0.0005) decreased the binding of the radiolabeled affibody molecules. The binding was proportional to EGFR expression, and binding to PC3 cells with low expression was the lowest.

The data concerning the binding affinity of three radiolabeled variants to A431 EGFR-expressing cell are presented in Table 4 and Supplemental Fig. 5. According to LigandTracer measurements, the best fit of the binding of the three radiolabeled variants to A431 cell line was achieved using a 1:2 model, suggesting that there were two types of interactions with EGF receptors, having almost equal association constants but different dissociation rates. All variants showed affinities in the low nanomolar and high picomolar ranges. The dissociation constants at equilibrium (*K*_D_) for the stronger interaction were between 72.8 and 340 pM, while for the second were between 2.2 and 8.03 nM.

**Table 4:** Equilibrium dissociation (*K*_D_) constants for the interaction between ^99m^Tc-labeled ZEGFR variants and EGFR-expressing A431 cells determined using an Interaction Map analysis of the LigandTracer sensorgrams

	Association rate constant (10^4^ 1/Ms)	Dissociation rate constant (10^−6^ 1/s)	*K*_D1_ (pM)	Weight (%)	Association rate constant (10^4^ 1/Ms)	Dissociation rate constant (10^−4^ 1/s)	*K*_D2_ (nM)	Weight (%)
ZEGFR-GGEC	8.2 ± 0.3	5.9 ± 0.8	73 ± 2	78	18.2 ± 0.2	5.5 ± 0.4	3.1 ± 0.9	22
ZEGFR-GEEC	4.5 ± 0.5	14.9 ± 0.8	334 ± 1	57	7.5 ± 0.2	7.1 ± 0.8	9.5 ± 0.1	43
ZEGFR-EEEC	11 ± 2	6 ± 1	66 ± 26	59	2 ± 1	4.3 ± 0.2	2.2 ± 0.04	41

**Fig. 3:**
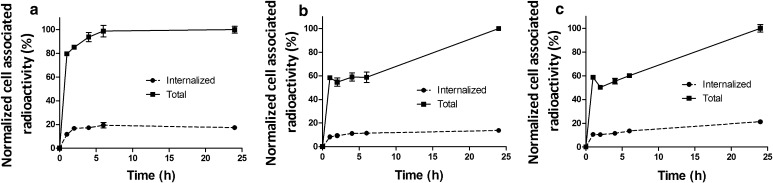
Cellular processing of ^99m^Tc-labeled ZEGFR (**a** ^99m^Tc-ZEGFR-GGEC, **b** ^99m^Tc-ZEGFR-GEEC and **c** ^99m^Tc-ZEGFR-EEEC) variants by EGFR-expressing A431 cells. Cells were incubated with 10 nM ^99m^Tc-labeled ZEGFR conjugate. The data are presented as average (*n* = 3) and SD. Error bars were not seen because they were smaller than point symbols

The processing of bound ^99m^Tc-ZEGFR variants by A431 cancer cell line is presented in Fig. 3. The common feature for the three labeled conjugates was rapid binding and a relatively low internalized fraction of ^99m^Tc-ZEGFR conjugates. The internalized fractions after 24 h incubation were 17.3 ± 0.5, 13.7 ± 0.3 and 21.2 ± 1% of total cell-bound radioactivity, for ^99m^Tc- ZEGFR-GGEC, ^99m^Tc-ZEGFR-GEEC and ^99m^Tc-ZEGFR-EEEC, respectively.

### In vivo studies

**Fig. 4:**
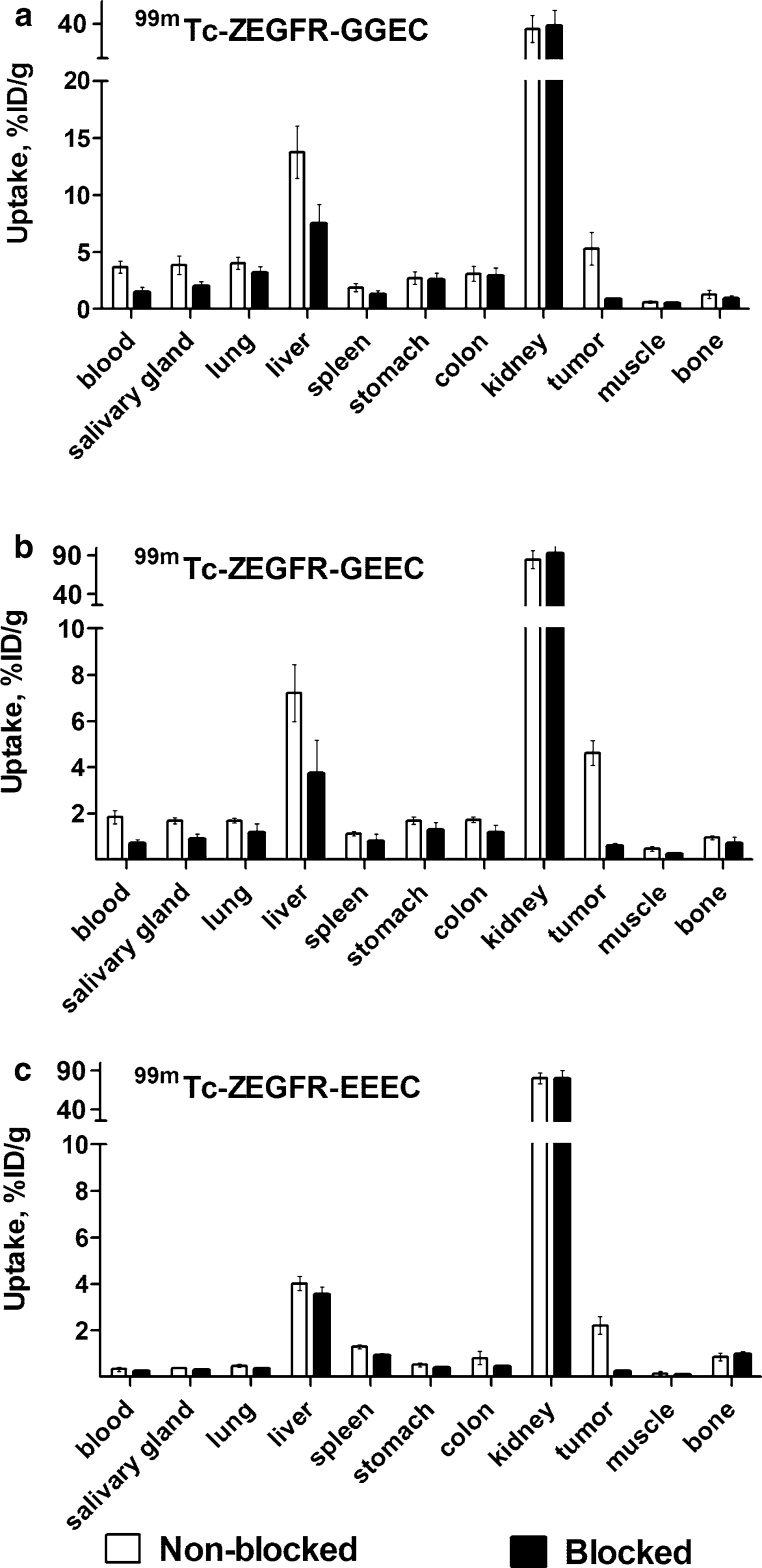
In vivo specificity of ^99m^Tc-ZEGFR conjugates (**a** ^99m^Tc-ZEGFR-GGEC, **b** ^99m^Tc-ZEGFR-GEEC and **c** ^99m^Tc-ZEGFR-EEEC) in A431 xenografts and EGFR-expressing organs in mice at 6 h after injection. In the blocked group, receptors were saturated by pre-injection of large excess of anti-EGFR antibody cetuximab. The data are presented as the average (*n* = 4) and SD

The in vivo specificity of ^99m^Tc-ZEGFR conjugates binding to EGFR was evaluated by pre-saturation of receptors by the monoclonal antibody cetuximab (Fig. 4). Tumor uptake in the blocked group was significantly (*P* < 0.05) reduced compared to non-blocked group for all three variants. For example, about 9-fold reduction in tumor uptake was observed for ^99m^Tc-ZEGFR-EEEC. For ^99m^Tc-ZEGFR-GGEC, significant (*P* < 0.05) reduction of radioactivity uptake in blood, salivary gland, liver, spleen, while for ^99m^Tc-ZEGFR-GEEC, significant (*P* < 0.05) reduction of radioactivity uptake in blood, salivary gland, liver, muscle and finally, for ^99m^Tc-ZEFGR-EEEC, significant (*P* < 0.05) reduction of radioactivity uptake in blood, salivary gland, lung, spleen, stomach and colon was observed.

**Fig. 5:**
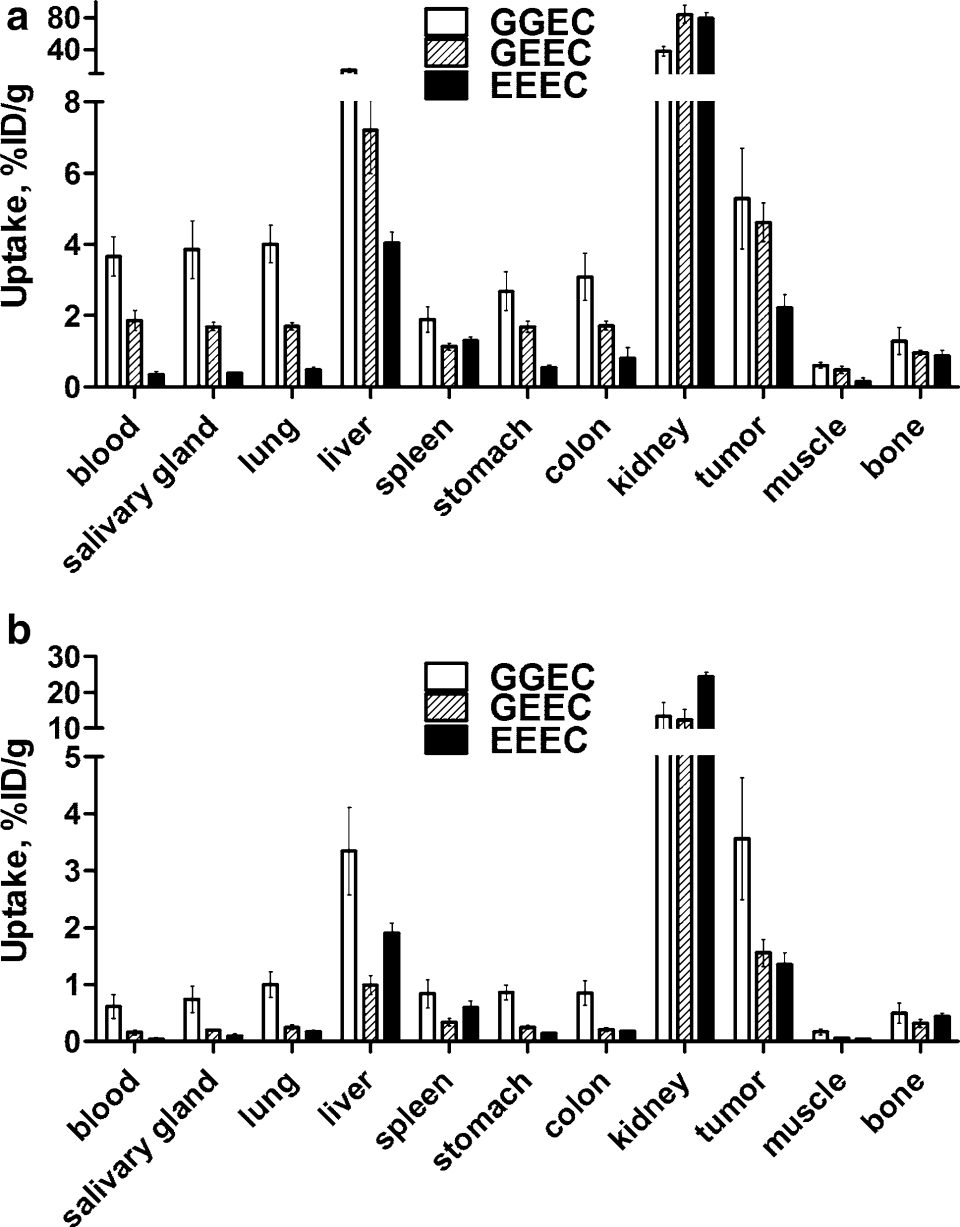
Biodistribution of ^99m^Tc-ZEGFR conjugates in BALB/C nu/nu mice bearing EGFR-expressing A431 xenografts at **a** 6 h and **b** 24 h after injection. The data are presented as the average (*n* = 4) and SD

**Fig. 6:**
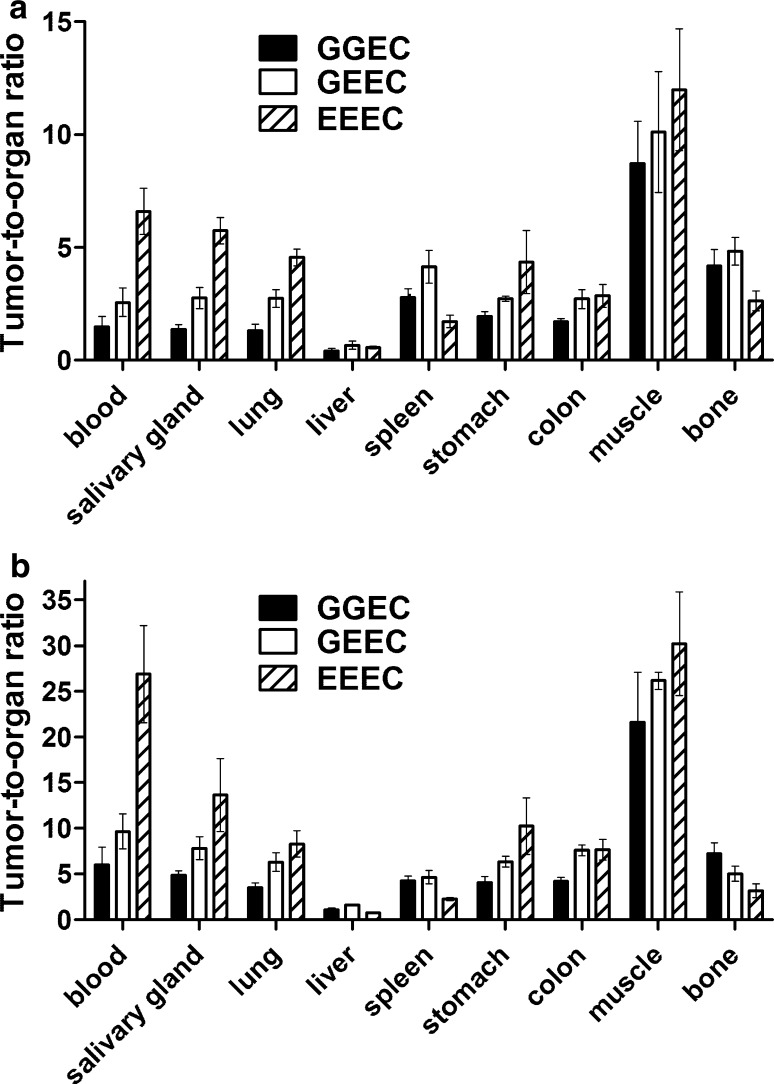
Tumor-to-organ ratios of ^99m^Tc-ZEGFR conjugates in BALB/C nu/nu mice bearing EGFR-expressing A431 xenografts at **a** 6 h and **b** 24 h after injection. The data are presented as the average (*n* = 4) and SD

Figure 5 shows the biodistribution of the ^99m^Tc-ZEGFR conjugates at 6 and 24 h after injection in BALB/C nu/nu mice bearing EGFR-expressing A431 xenografts. There were substantial differences in the biodistribution profiles of the affibody molecules labeled using different N_3_S chelators. ^99m^Tc-ZEGFR-GEEC and ^99m^Tc-ZEGFR-EEEC demonstrated the highest radioactivity accumulation (84 ± 11 and 80 ± 7% ID/g) in the kidneys at 6 h after injection. Tumor uptake values for ^99m^Tc-ZEGFR-GGEC, ^99m^Tc-ZEGFR-GEEC and ^99m^Tc-ZEGFR-EEEC at 6 h after injection were 5.3 ± 1.4, 4.6 ± 0.5 and 2.2 ± 0.4 %ID/g, respectively, and exceeded uptake in other organs except liver and kidneys. However, the tumor-to-organ ratios (Fig. 6) were modest at this time-point. Significant (*P* < 0.05) decrease of uptake in almost all other organs and tissues for three variants at 24 h compared to 6 h resulted in a profound increase of tumor-to-organ ratios (Fig. 6). For example, tumor-to-blood ratio increased 4-fold and tumor-to-liver, tumor-to-lung, tumor-to-muscle ratios increased 2–4-fold. Additionally, the renal uptake of ^99m^Tc-labels decreased 3–7-fold, although tumor-to-kidney ratios remained less than one, 0.27 ± 0.02, 0.13 ± 0.01, and 0.05 ± 0.01 for ^99m^Tc-ZEGFR-GGEC, ^99m^Tc-ZEGFR-GEEC and ^99m^Tc-ZEGFR-EEEC, respectively. This indicated that the residualizing properties of ^99m^Tc-labels are moderate, and radiometabolites ‘leak’ from cells after internalization. It is likely that this is the reason why ^99m^Tc clears more rapidly from other normal tissues.

^99m^Tc-ZEGFR-EEEC showed the highest tumor-to-organ ratio for salivary gland, lung, stomach, colon and muscle in all the studied time-points. Additionally, this ^99m^Tc-labeled affibody molecule showed the highest tumor-to-blood ratio at 6 h (6.6 ± 1) and 24 h (26.9 ± 5.3). This suggested that better contrast of imaging at later time-points and should provide better sensitivity of clinical imaging at 24 h after injection. In terms of tumor-to-liver ratio, ^99m^Tc-ZEGFR-GEEC showed the highest values at both time points. Additionally, tumor-to-liver, tumor-to-lung, tumor-to-muscle ratios increased ~ 2–4-fold after 24 h after injection.

**Fig. 7:**
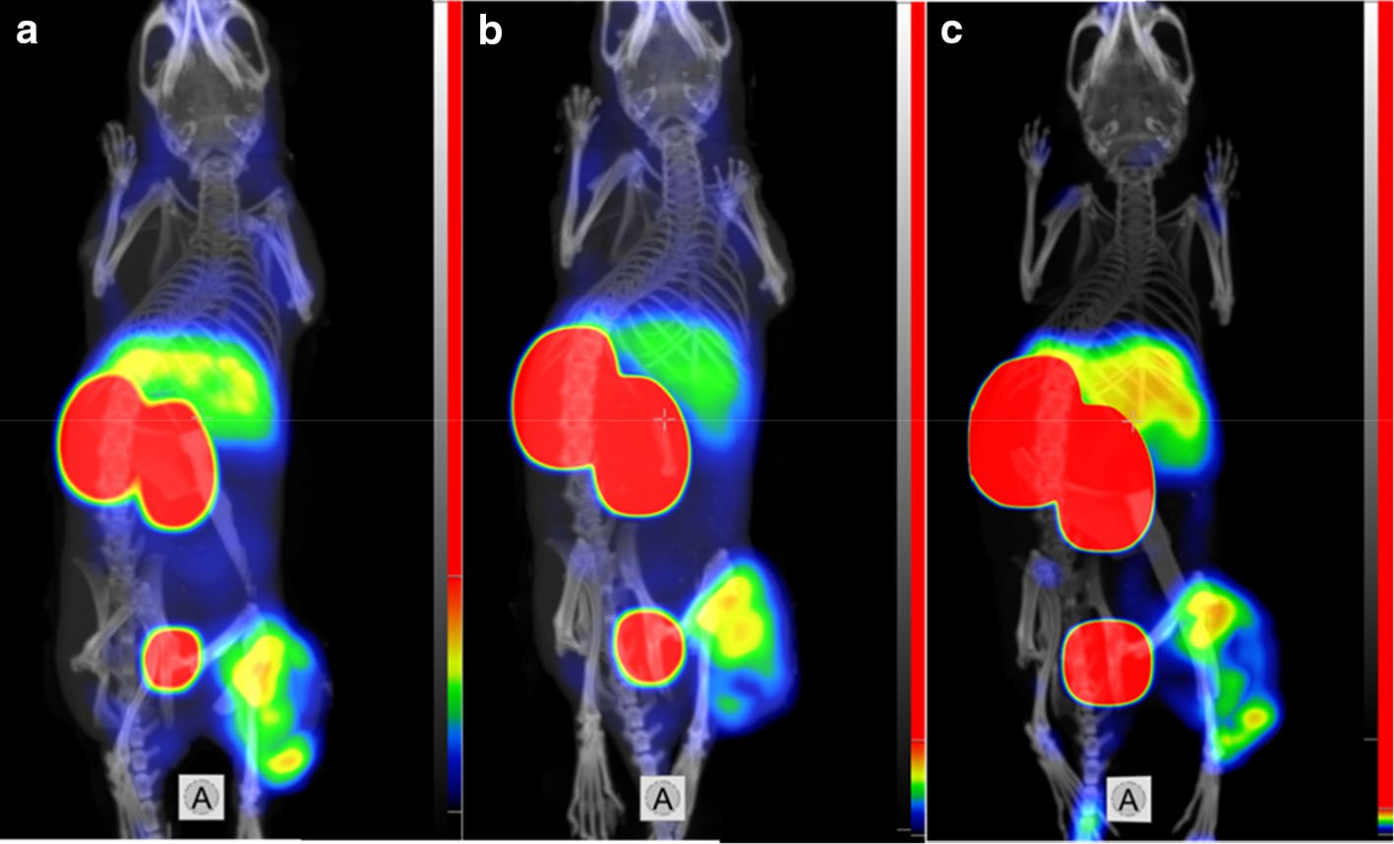
Imaging of EGFR-expressing A431 xenografts in BALB/C nu/nu mice using **a** ^99m^Tc-ZEGFR-GGEC, **b** ^99m^Tc-ZEGFR-GEEC and **c** ^99m^Tc-ZEGFR-EEEC at 6 h after injection. The scales are adjusted to first red pixels in tumors

**Fig. 8:**
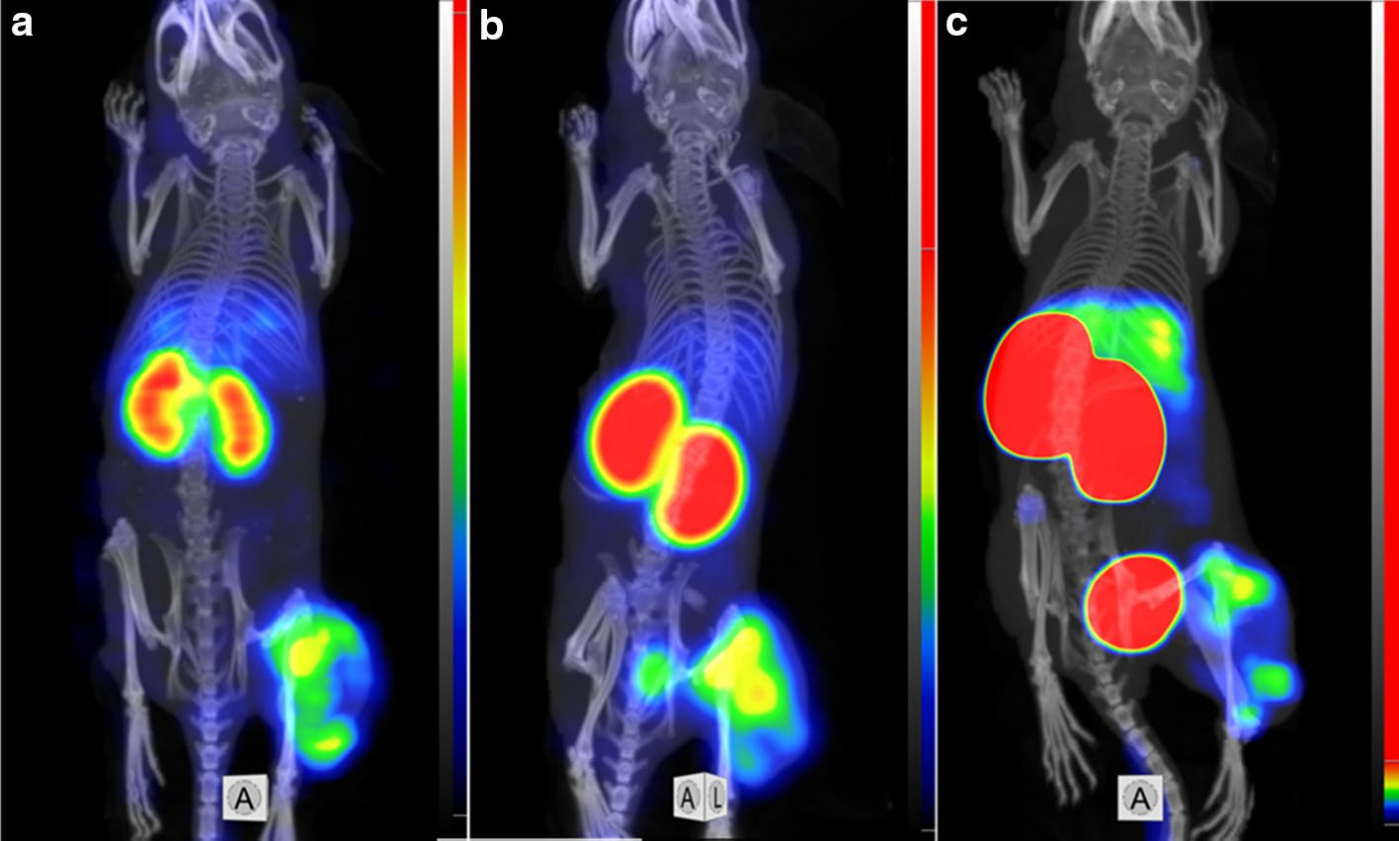
Imaging of EGFR-expressing A431 xenografts in BALB/C nu/nu mice using **a** ^99m^Tc-ZEGFR-GGEC, **b** ^99m^Tc-ZEGFR-GEEC and **c** ^99m^Tc-ZEGFR-EEEC at 24 h after injection. The scales are adjusted to first red pixels in tumors

Results of microSPECT/CT imaging (Figs. 7, 8) demonstrated that visualization of EGFR expression in tumors using affibody molecules labeled with ^99m^Tc using peptide-based glutamate-containing chelators is feasible. The tumors on hind legs were clearly visualized at both studied time-points, although the tumor uptake of the tracers was heterogeneous. In agreement with the biodistribution data, the radioactivity uptake in kidneys was considerably higher than in tumor. Besides tumors and kidneys, liver was the only place with noticeable radioactivity accumulation. ^99m^Tc-ZEGFR-GEEC provided higher tumor-to-liver ratio than other variants. The contrast to liver was increased at 24 h after injection.

## Discussion

Previous studies have demonstrated that a translation of methodology for ^99m^Tc-labeling to new affibody molecules is possible (Mitran et al. 2015; Andersson et al. 2016). However, re-optimization of labeling conditions is required to obtain the desirable results since the composition of amino acids in the binding site or in the scaffold of affibody molecules might have a profound influence on the site-specificity of labeling as well as on the stability of the label (Lindberg et al. 2012; Mitran et al. 2015; Andersson et al. 2016). Indeed, the use of Protocol A resulted in an appreciable release of ^99m^Tc from all conjugates (except form ^99m^Tc-ZEGFR-GEEC) even after simple dilution with PBS and under cysteine challenge (Fig. 2). Adding anti-oxidants such as sodium ascorbate or tin (II) chloride resulted in a remarkable reduction in ^99m^Tc release. This indicated that the main reason of the release was re-oxidation of technetium. This was not what we have observed earlier for anti-HER2 and anti-IGF-1R affibody molecules, where even -GGGC provided a stable attachment of ^99m^Tc and ^188^Re (Wållberg et al. 2011; Altai et al. 2012, 2014a, b; Mitran et al. 2015). A possible explanation for the current results is that some side chains in the affibody molecules form an alternative chelating pocket and a part of technetium is bound not only to a cysteine-containing chelator, but also to this much weaker site.

We have found that the use of a pre-purification challenge (Protocol B) resulted in reduced radiochemical yield (Table 3), but the radioactivity loss during stability tests was remarkably lower than after labeling according to Protocol A (Fig. 2). This indicates that the use the pre-purification challenge removes technetium that was loosely bound to the affibody molecules and improves stability of the purified conjugate.

The effect of amino acids with electron donating side chains on stability of ^99m^Tc-complex was quite obvious. For example, ^99m^Tc -ZEGFR-GGGC and ^99m^Tc-ZEGFR-APKC showed the lowest labeling and isolated yields (Protocol B), and the lowest stability. ^99m^Tc -ZEGFR-GGEC and ^99m^Tc-ZEGFR-GEEC variants, which contained electron-donating glutamates in the chelators, showed the best labeling and stability. Interestingly, ^99m^Tc-ZEGFR-EEEC did not follow this trend. It is possible that the glutamate, which is the most distant from the cysteine, creates a steric hindrance for optimal complex geometry. Taking together, the use of glutamate-containing chelators and the pre-purification challenge was a reasonable strategy for improving yield and stability of ^99m^Tc-labeled anti-EGFR affibody molecules.

Blocking of receptors on EGFR-expressing cells with anti-EGFR antibody cetuximab reduced significantly (*P* < 0.005) binding of ^99m^Tc-ZEGFR conjugates, which confirmed specificity (Supplemental Fig. 4). Kinetics of ^99m^Tc-ZEGFR-GGEC, ^99m^Tc-ZEGFR-GEEC and ^99m^Tc-ZEGFR-EEEC binding to A431 cells was best fitted to an interaction model with two binding sites, one having subnanomolar and another having low nanomolar affinity (Table 3; Supplemental Fig. 5). Presence of two types of interactions has earlier been described for several natural and artificial binders to dimerizing receptor tyrosine kinases (Bjorkelund et al. 2011; Barta et al. 2012; Tolmachev et al. 2012). Bjorkelund and co-workers (2011) have demonstrated that the presence of a second affinity of interaction for epidermal growth factor with EGFR is associated with the receptor dimerization and is, most likely, caused by dimerization-related conformational changes. Therefore, it is possible that the binding of the ^99m^Tc-labeled affibody conjugates is also sensitive to slight conformational changes of the epitope due to homo- and heterodimer formation by EGFR.

Cellular processing of the three variants reveals that there is a relatively low percentage of internalized fractions, which is typical for monomeric affibody molecules (Fig. 3). The total bound radioactivity for ^99m^Tc-ZEGFR-GEEC and ^99m^Tc-ZEGFR-EEEC showed an initial phase with a more rapid increase followed by a second phase with slower increase over time, while a rapid increase followed by plateau was observed for ^99m^Tc-ZEGFR-GGEC. Possible reasons for these results might be that ^99m^Tc-ZEGFR-GEEC and ^99m^Tc-ZEGFR-EEEC have stronger residualizing properties compared to ^99m^Tc-ZEGFR-GGEC.

The in vivo receptor saturation experiment demonstrated that all tested conjugates accumulated specifically in EGFR-expressing A431 xenografts (Fig. 4). Remarkably, the in vivo binding specificity was preserved also for ^99m^Tc-ZEGFR-EEEC despite its low melting point. In addition, there was significant reduction in EGFR-expressing tissues (e.g. liver and salivary glands) although the effect was not as pronounced because of limited binding of cetuximab to murine EGFR.

There was a pronounced effect of the glutamate content in the chelators on the biodistribution of affibody molecules. One clear example is the renal retention of radioactivity. After glomerular filtration, affibody molecules are re-absorbed in the proximal tubuli of kidneys and rapidly internalized (Feldwisch and Tolmachev 2012). Thus, the renal retention of a radionuclide depends on its residualizing properties. In this study, radioactivity accumulation in kidneys was lowest for the ^99m^Tc-ZEGFR-GGEC. This is in agreement with the data on low renal retention of radioactivity in the case of anti-HER2 affibody molecules labeled with ^99m^Tc using the same chelator (Wållberg et al. 2011). This observation correlates with limited residualizing properties indicated by the cellular processing studies. Elevated retention of renal radioactivity in the case of ^99m^Tc-ZEGFR-EEEC corresponds with an earlier observation that increase of glutamate content in mercaptoacetyl-containing peptides-based chelators located at N-terminus of synthetic affibody molecules results in stronger residualizing properties and higher retention of radioactivity in kidneys (Tran et al. 2007).

A challenge for in vivo imaging of EGFR is the EGFR expression in liver (Divgi et al. 1991). Hepatocytes express around 600,000 receptors per cell (Benveniste et al. 1988). Since internalization of affibody molecules after binding to EGFR is slow (Tolmachev et al. 2010; Garousi et al. 2016, 2017), radiolabeled affibody molecules remain reversibly bound to the surface of hepatocytes and dissociate when blood concentration is decreased because of renal clearance. Hence, liver acts as depots for anti-EGFR affibody molecules. This results in slower blood clearance compared with other affibody molecules and the highest contrast is also reached at a later time point. Furthermore, we have found that the hepatic uptake of anti-EGFR affibody molecules is most likely mediated by two different mechanisms. One mechanism is EGFR-dependent and can be saturated. The second mechanism is independent on EGFR-binding, but depends on both charge and charge distribution in the C-terminus of affibody molecules (Garousi et al. 2017). Data from this study suggest that the second mechanism plays a crucial role since the hepatic uptake is reversely proportional to the number of glutamates in the chelators (Supplemental Fig. 6a). The reduction of hepatic uptake with increase of hydrophilicity and negative charge is in agreement with multiple observations for peptides and scaffold proteins (Hosseinimehr et al. 2012).

Increase of number of glutamates in the chelating site of anti-ZEGFR conjugate was associated with more rapid clearance from blood (Supplemental Fig. 5b). Earlier, similar results have been observed for anti-HER2 affibody molecules labeled with ^99m^Tc at the N-terminus using peptide-based mercaptoacetyl-containing chelators (Tran et al. 2007). This is also in agreement with the observation reported by Rusckowski and co-workers (2001) that transient binding of labeled peptides to blood proteins is due to presence of “hydrophobic patches” and might be counteracted by the use of more hydrophilic chelators. Furthermore, uptake in lung, salivary gland, stomach, colon and muscle at 6 h after injection correlated strongly with radioactivity concentration in blood (Supplemental Fig. 7a-d). Tumor uptake increased also along with higher concentration in blood (Supplemental Fig. 7f), which is most likely due to a somewhat better bioavailability. However, this dependence was not as pronounced as for normal tissues, which resulted in appreciable increase in tumor-to-tissue ratios with the increase of number glutamate in the chelators. Therefore, ^99m^Tc-ZEGFR-EEEC provided the highest tumor-to-organ ratios.

The use of chelator engineering enabled appreciable improvement of tumor-to-organ ratios compared with the homologous tracer with a peptide-based chelator obtained by adding a cysteine to a wild-type C-terminus (Andersson et al. 2016). ^99m^Tc-ZEGFR-EEEC provided ca. 3-fold higher tumor-to-blood and tumor-to-salivary gland ratios, ca. 2-fold higher tumor-to-lung and tumor-to-colon ratios and 1.6-fold higher tumor-to-muscle ratio.

In conclusion, the use of peptide-based cysteine-containing chelators for labeling of anti-EGFR affibody molecules with ^99m^Tc is possible. Re-optimization of labeling procedure might be necessary for transfer of previously established labeling methodology to new affibody molecules. The use of glutamate-containing chelators improves stability of coupling of the nuclide. Increase of glutamate content in peptide-based chelators enhances contrast of affibody-mediated imaging.

## Electronic supplementary material

Below is the link to the electronic supplementary material.

Supplementary material 1 (DOCX 808 kb)

## Abbreviations

EGFR: Epidermal growth factor receptor
HER2: Human epidermal growth factor receptor type 2
HER3: Human epidermal growth factor receptor type 3
IGF-1R: Insulin-like growth factor 1 receptor
CAIX: Carbonic anhydrase IX
SPECT: Single photon emission computed tomography
PET: Positron emission tomography
ITLC: Instant thin-layer chromatography
SDS PAGE: Sodium dodecyl sulfate polyacrylamide gel electrophoresis
