# Supplementary material for: Influence of composition of cysteine-containing peptide-based chelators on biodistribution of 99mTc-labeled anti-EGFR affibody molecules
Source: Amino Acids. 2018 May 4;50(8):981–94. doi: 10.1007/s00726-018-2571-1 (PMC6060960; doi:10.1007/s00726-018-2571-1)
Supplement: Supplementary file 1 — Supplementary material 1 (DOCX 808 kb) [file 726_2018_2571_MOESM1_ESM.docx]

**Supplementary material**

Maryam Oroujeni^1*^, Ken G Andersson^2*^, Xenia Steinhardt^2^, Mohamed Altai^1^, Anna Orlova^3^, Bogdan Mitran^3^, Anzhelika Vorobyeva^1^, Javad Garousi^1^, Vladimir Tolmachev^1^, John Löfblom ^2^.

**Influence of composition of cysteine-containing peptide based chelators on biodistribution of ^99m^Tc-labeled anti-EGFR affibody molecules**

^1^Department of Immunology, Genetics and Pathology, Uppsala University, Uppsala, Sweden;

^2^Department of Protein Science, KTH - Royal Institute of Technology, Stockholm, Sweden;

^3^Department of Medicinal Chemistry, Uppsala University, Uppsala, Sweden;

**Corresponding author**

Vladimir Tolmachev.

Department of Immunology, Genetics and Pathology,

Uppsala University,

SE-75181,

Uppsala,

Sweden;

Tel. +46 18 471 34 14

Mobile: +46 704 250782

e-mail: [vladimir.tolmachev@igp.uu.se](mailto:vladimir.tolmachev@igp.uu.se)

ORCID: 0000-0002-6122-1734


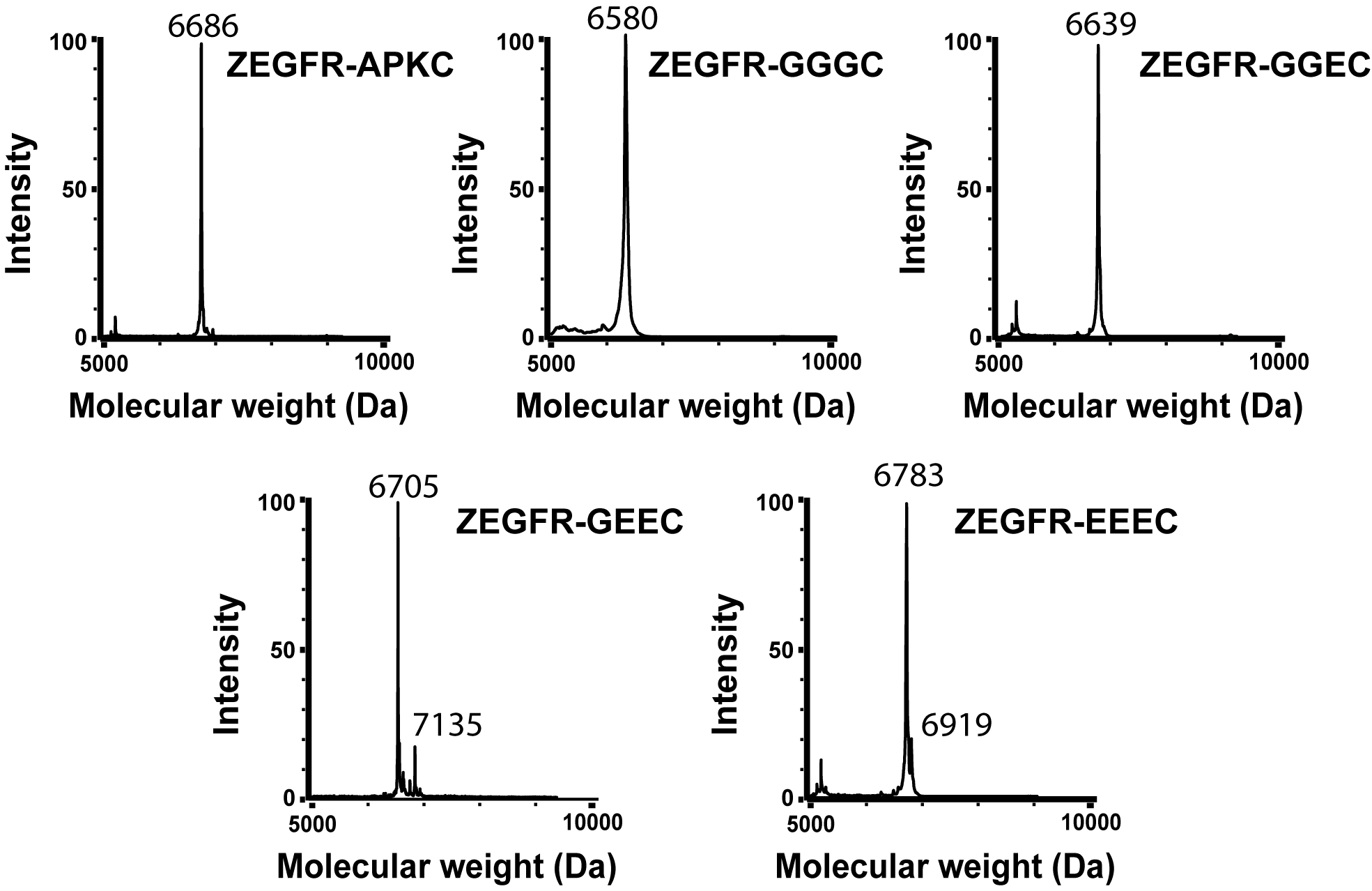


**Supplemental Fig. 1** Mass-spectra of affibody molecules.


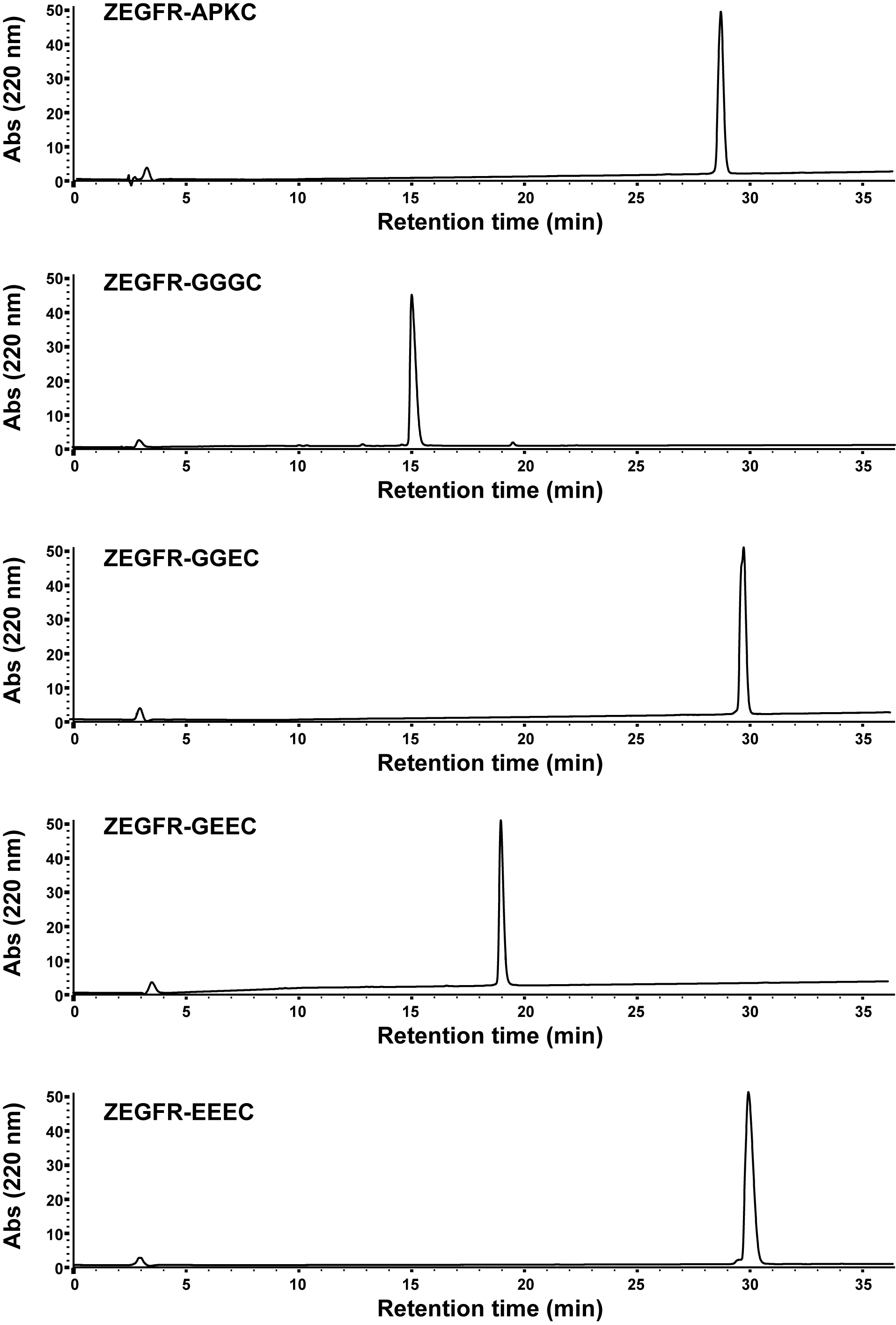


**Supplemental Fig. 2** Analytical HPLC chromatograms of affibody molecules. The constructs GGGC and GEEC were analyzed in a gradient from 40-50 % during 35 minutes, while the other three constructs were analyzed in a 30-50% gradient during 35 minutes.


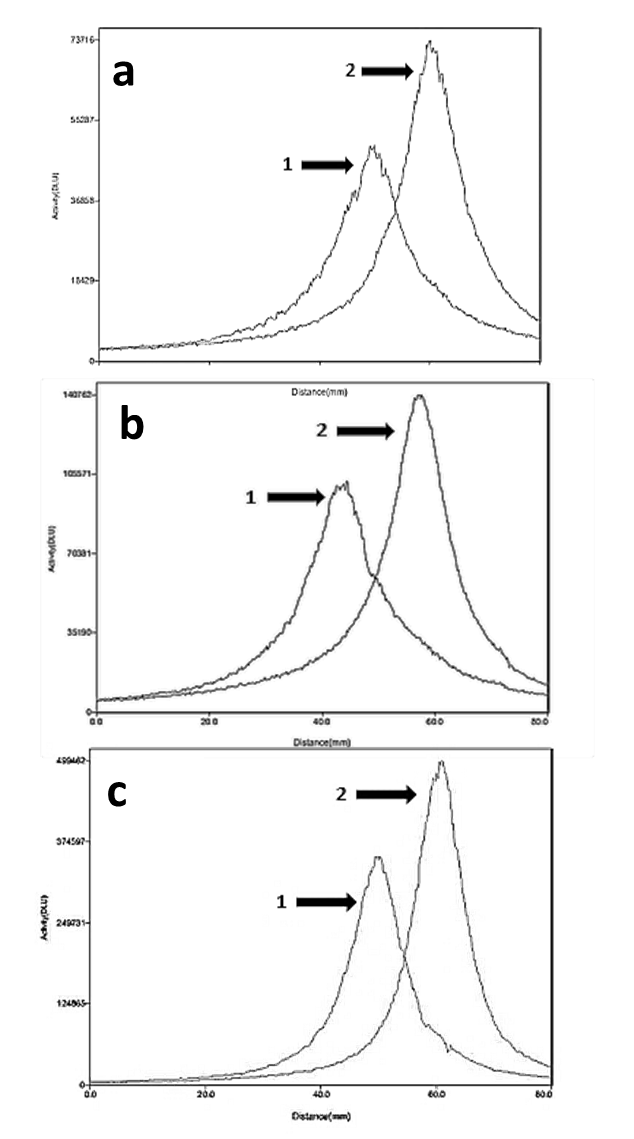


**Supplemental Fig. 3** SDS-PAGE analysis of ^99m^Tc-ZEGFR-GGEC (a), ^99m^Tc-ZEGFR-GEEC (b) and ^99m^Tc-ZEGFR-EEEC (c). Distribution of radioactivity along lanes was visualized and quantified using Cyclone™ Storage Phosphor System. **1** radiolabeled affibody molecule sample; 2.^99m^Tc-pertechnetate was used as a marker for low molecular weight compounds

**Supplemental Fig. 4** In vitro specificity of three ^99m^Tc labelled ZEGFR variants binding (a: ^99m^Tc-ZEGFR-GGEC, b: ^99m^Tc-ZEGFR-GEEC and c: ^99m^Tc-ZEGFR-EEEC) to three different EGFR-expressing cell lines. Cells were incubated with 10 nM ^99m^Tc labelled conjugate. 500 nM cetuximab was used for blocking of the receptors. The data are presented as the average (n=3) and SD


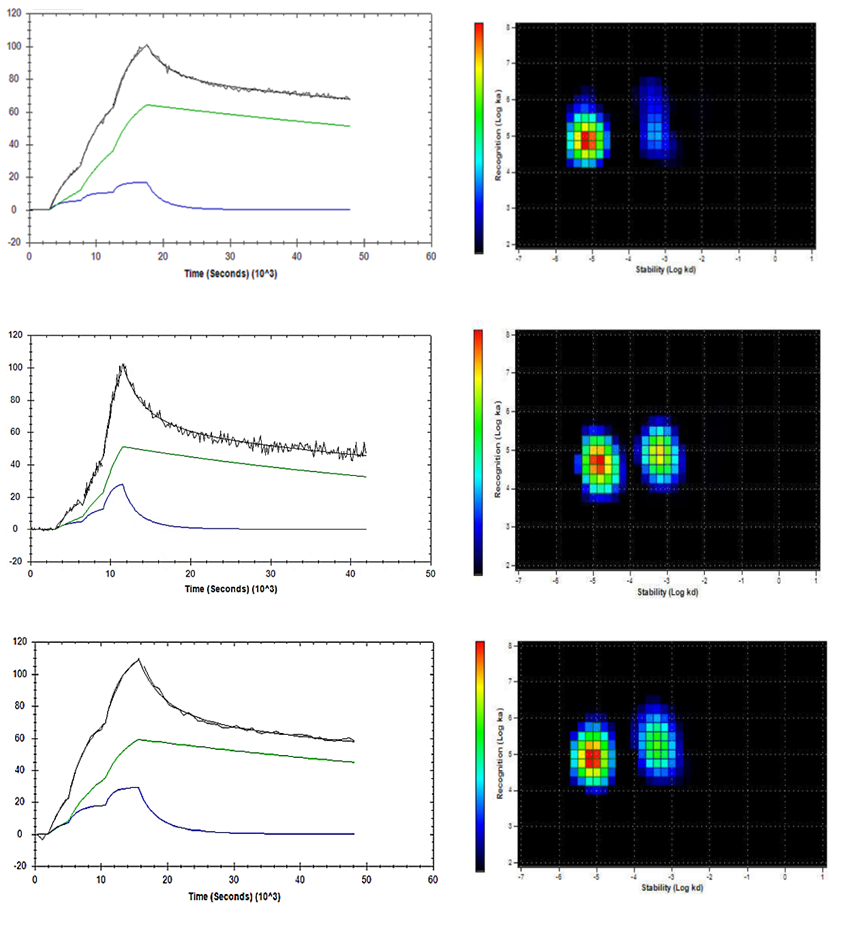


**Supplemental Fig. 5** Sensorgrams (left) and InteractionMaps (right) describing binding of ^99m^Tc-labeled affibody molecules to EGFR-expression A431 cells.( a) ^99m^Tc-ZEGFR-GGEC; (b) ^99m^Tc-ZEGFR-GGEC; (c) ^99m^Tc-ZEGFR-EEEC. The colors correspond to the degree of contribution, where warmer colors representing larger contributions. The data show two interactions with similar association rate (k_a_), but with different dissociation rates (k_d_).

**Supplemental Fig. 6** Influence of the number of glutamates in the chelator on uptake of ^99m^Tc-labeled affibody molecules in liver (a) and concentration in blood (b) 6 h after injection.

**Supplemental Fig. 7** Correlation between concentration of 99mTc-labeled affibody molecules in blood and uptake in lung (a), salivary gland (b), stomach (c), colon (d), muscle (e) and tumor (f).
